# Provision of oral healthcare services in WHO-EMR countries: a scoping review

**DOI:** 10.1186/s12903-024-04446-9

**Published:** 2024-06-18

**Authors:** Lamis Abuhaloob, Celine Tabche, Federica Amati, Salman Rawaf

**Affiliations:** WHO Collaborating Centre for Public Health Education and Training, Primary Care & Public Health, School of Public Health, Faculty of Medicine, Imperial College London, The Reynolds Building, St. Dunstan’s Road, London, W6 8RP UK

**Keywords:** Dental health performance assessment, Primary health care, WHO-Eastern Mediterranean Region

## Abstract

Global neglect of oral healthcare services (OHCS) provision, mainly in Low- and Middle-Income Countries, exacerbates the deterioration of health systems and increases global health inequality.

Objectives

The objective is to explore the profiles of available oral healthcare services in the WHO Eastern Mediterranean Region (EMR) countries.

Methods

A systematic literature search was conducted of grey literature and databases (PubMed, Medline, Embase, and the Cochrane Library). Peer-reviewed articles that reviewed and/or evaluated OHCS in WHO-EMR countries were identified. No time or language limitations were applied. Two independent reviewers conducted the screening and data extraction. A third reviewer arbitrated disagreement. The evaluation of the OHCS provision followed the WHO framework for health system performance assessment. The extraction included socio-demographic characteristics of the studied population, OHCS profile, responsiveness, and health insurance coverage.

Results

One hundred and thirty-seven studies were identified. The studies that met the inclusion criteria were fifteen published between 1987 and 2016. In addition, two reports were published in 2022. The included studies were conducted in Pakistan, Saudi Arabia, Iran, Libya, Egypt, Oman, Syria, Jourdan, Kuwait, and Tunisia. Generally, Ministries of Health are the main providers of OHCS. The provision for national dental care prevention programmes was highly limited. Furthermore, most of these Ministries of Health have struggled to meet their local populations’ dental needs due to limited finances and resources for OHCS.

Conclusions

Oral and dental diseases are highly prevalent in the WHO-EMR region and the governments of the region face many challenges to meeting the OHCS needs of the population. Therefore, further studies to assess and re-design the OHCS in these countries to adapt dental care prevention into national health programmes are crucial.

## Background

The most recent global WHO survey reported a lack of preventive oral health programmes in Low- and Middle-Income Countries (LMICs). Moreover, the same report shows a scarcity of information on oral healthcare services (OHCS) in the WHO Eastern Mediterranean Region (WHO-EMR) [1]. Furthermore, other recent reviews also highlighted the same shortage of preventative oral healthcare programmes in the region [2, 3]. Internationally, there is a growing consensus that neglect of oral health prevention and services [4, 5], lack of dental public health education, and growing political conflict negatively affect the population's dental health. Consequently, the risks of deterioration of oral health, services, and health equality are increasing.

As an example of the destructive impact of long-lasting armed conflicts on oral healthcare, the persistent conflict in the Occupied Palestinian Territories has produced a severe deterioration of health, oral health, and social care services [6–8]. As a result of the financial restrictions affecting existing health services, local governments and international bodies were unable to meet healthcare needs and promote overall oral health [9–12].

In addition, previous studies found deficiencies in the availability and effectiveness of oral health interventions aimed at promoting children and maternal oral health in LMICs [2, 3, 5]. The United Nations Relief and Works Agency (UNRWA) reported increased oral health impairments among Palestinian refugees living in the Occupied Palestinian Territories and the surrounding countries (Jordan, Syria, and Lebanon) [13]. Oral health surveys conducted in Jordan [14, 15], Syria [16] and Lebanon [17] highlighted the same poor oral health status among Indigenous populations. Besides that, the situation in other Middle Eastern countries does not show any better oral health profiles [18, 19], even in high-income countries such as the Arab Gulf states [20]. The most recent report published by the WHO demonstrated a high deficiency in the contribution of the national health budget to OHCS. In addition, there was a lack of preventive programmes in these countries associated with a high prevalence of untreated decayed teeth, mainly among children [5].

Therefore, the necessity of empowering OHCS and integrating the evidence-based WHO oral health promotion programmes into ongoing healthcare programmes should be a priority for policy makers in the Region [5, 12, 21].

To achieve that, there is still a need to explore the profile of current oral healthcare systems in WHO-EMR countries, which include 19 out of the 22 Arab countries, at least eight of which are in conflict zones. This information would help assess the performance and efficacy of promoting oral health in the region. In addition, it would enable healthcare stakeholders and scientists to investigate if the ongoing OHCS in LMICs including conflict zones could adapt the WHO oral health promotion interventions while maintaining high performance and outcomes to reduce oral health inequalities [21].

Unfortunately, such information is not currently available to answer these questions. The information is essential to identify factors, gaps, and challenges influencing the performance of OHCS. Moreover, it will help to envisage OHCS's capability to adapt the WHO oral health promotion interventions and maintain high performance. Therefore, the main aim of this scoping review is to explore the profiles of OHCS in the WHO-EMR countries and to evaluate their performance.

## Methods

### Research questions formulation

This scoping review was conducted to determine the profiles of OHCS in the WHO-EMR countries, evaluate their performance, and identify the gaps and challenges for OHCS in these countries.

Therefore, the main research questions are ‘What is the profile of the provided OHCS in the countries of the WHO-EMR?’ and ‘Do these services perform effectively to promote the oral health of populations?’

Additionally, ‘What factors, gaps, and challenges influence this performance?’ and ‘What is the capability of OHCS to adapt the WHO oral health promotion interventions and maintain its high performance?’

### Determining the search strategy

A literature search was conducted using the following databases: MEDLINE, Embase, and the Cochrane Library. Furthermore, the websites of the Ministries of Health in WHO-EMR countries, the World Health Organization (WHO), the International Union for Health Promotion and Education (IUHPE), the United Nations Children's Fund (UNICEF), United Nations Relief and Works Agency for Palestine Refugees in the Near East (UNRWA), and the United Nations Refugee Agency (UNHCR) were searched.

Search terms were investigated, including: 1 “exp refugee/ or UNRWA.mp.”, 2 “Eastern Mediterranean*.mp.”, 3 “Middle East*.mp.”, 4 “Afghanistan.mp.”, 5 “Bahrain.mp.”, 6 “Djibouti.mp.”, 7 “Egypt.mp.”, 8 “Iran.mp.”, 9 “Iraq.mp.”, 10 “Jordan.mp.”, 11 “Kuwait.mp.” 12 “Lebanon.mp.”, 13 “Libya.mp.”, 14 “exp Palestine/ or Occupied Palestinian territory.mp.”, 15 “Morocco.mp.”, 16 “Oman.mp.”, 17 “Pakistan.mp.”, 18 “Qatar.mp.”, 19 “Saudi Arabia.mp.”, 20 “Somalia.mp.”, 21 “Sudan.mp.”, 22 “Syria.mp. or exp Syrian Arab Republic/”, 23 “Tunisia.mp.”, 24 “United Arab Emirates.mp.”, 25 “Yemen.mp.”, 26 “Algeria.mp.”, 27“WHO EMRO*.mp.”, 28 “1 or 2 or 3 or 4 or 5 or 6 or 7 or 8 or 9 or 10 or 11 or 12 or 13 or 14 or 15 or 16 or 17 or 18 or 19 or 20 or 21 or 22 or 23 or 24 or 25 or 26 or 27”, 29“*dental care/ or Oral Health*.mp.”, 30 “*health care delivery/ or health system*.mp. or *health care system/”, 31 “*primary health care/”, 32 “*total quality management/”, 33 “*dental health/ or *dental care/ or Dental Health*.mp.”, 34 “29 or 33”, 35 “30 or 31 or 32”, 36 “34 and 35”, 37 “28 and 36”.

The search was conducted between 15 Jan 2022 and 30 Jan 2023. No limitations for the period or language of studies were specified. All eligible papers determined after the first screening were reviewed ensuring that each paper met the inclusion and exclusion criteria (Table 1).

**Table 1:** Inclusion and exclusion criteria

**Inclusion Criteria**	**Exclusion Criteria**
**Population:** Population living in WHO-EMR countries (Indigenous and/or refugees) **Type of Studies:** Original articles, systematic reviews, and oral healthcare information published in the reports of local ministries’ health, UNRWA and/or WHO websites. **Intervention / Exposure:** The oral healthcare interventions operated within the WHO-EMR countries and their performance. **Outcomes:** The primary outcome is investigating the availability of OHCS and the effectiveness of their performance. The evaluation of oral health system performance followed the WHO framework, which declares that an effective healthcare service should achieve the main three cross-goals for the health system (Murray et al., 1999): health (oral health), responsiveness, and fairness in financing. The level of effectiveness of OHCS in improving oral healthcare was measured by assessing the change in oral health indicators over time, either as a result of the implementation of OHCS, Randomised Control Trials, or interventions (change in the incidence of dental caries and/or experience; the incidence of gingivitis and periodontitis; and percentage of people receiving preventive/curative OHCS). Change in responsiveness indicators (respect percentage for a person’s dignity, autonomy, confidentiality, and satisfaction over the years) were included. Moreover, the shift in fairness in financing indicators (change in percentage of OHCS covered by the health insurance and national health budget contribution into oral healthcare expenditure (percentage)) was also included. The secondary outcome was identifying factors, gaps, and challenges influencing the performance of OHCS and foreseeing its capability to maintain high oral healthcare performance. **Additional outcome:** The secondary outcome was identifying factors, gaps, and challenges influencing the performance of OHCS in the EMRO region and foreseeing its capability to adapt the WHO oral health promotion interventions and maintain high oral healthcare performance.	**Population:** Populations and/or refugees not living in WHO-EMR countries **Intervention / Exposure:** Studies not evaluating OHCS and/or their performance and outcome. **Study outcome:** Not recording any information for the OHCS and/or no outcome regarding OHCS performance Study full text not found.

### Screening: Selecting the literature

**Fig. 1:**
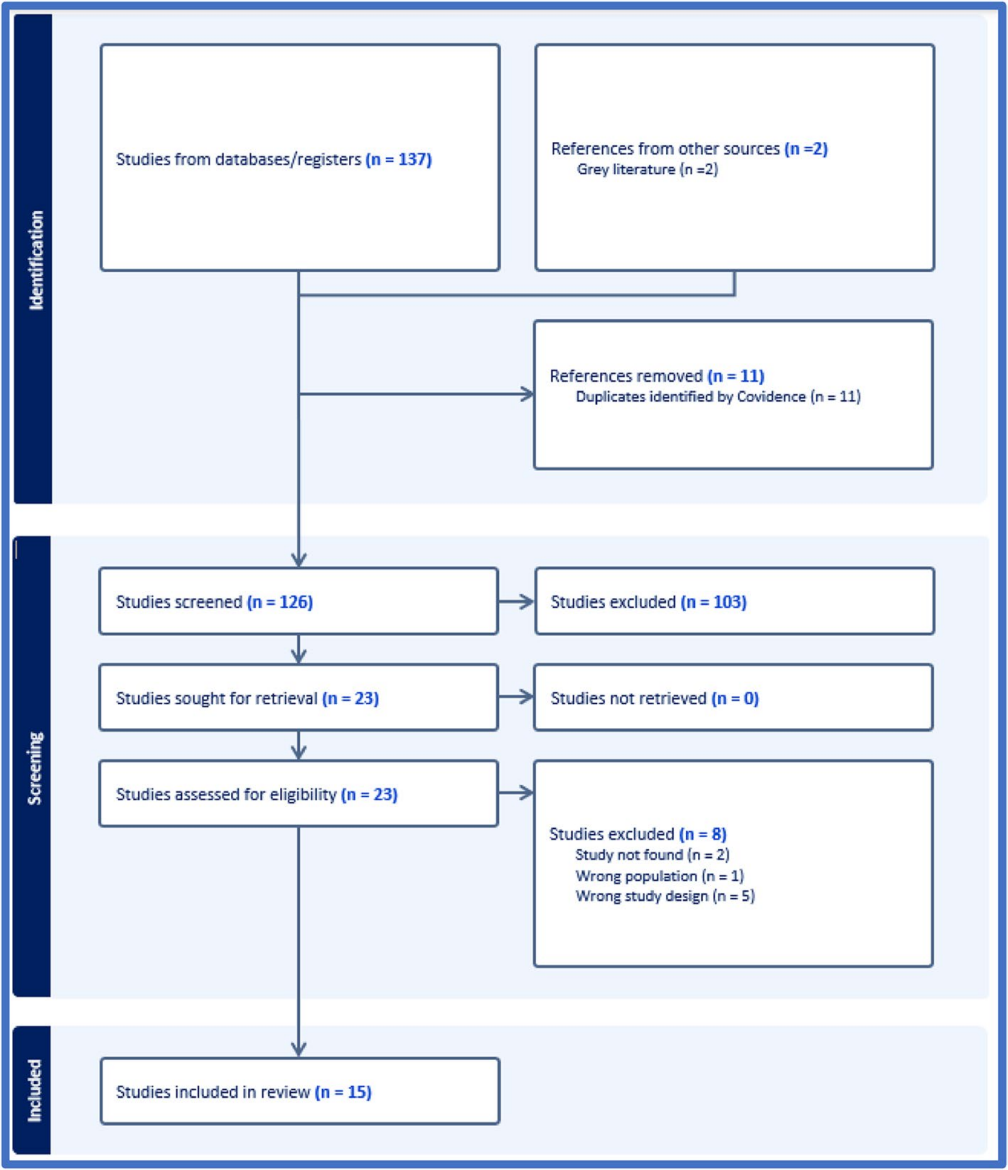
Prisma chart for studies screening and inclusion [20]

Two independent researchers selected the studies relevant to the research question. The two independent researchers screened the title and abstracts. After that, they independently reviewed the full text obtained to identify the included studies, as detailed in the PRISMA flow diagram in Fig. 1. The third reviewer arbitrated disagreement on study inclusion between the two reviewers.

The current review explored the profiles of OHCS in WHO-EMR countries. A systematic search was conducted for relevant databases and grey literature. Two reports were identified from the grey literature: [1] the OHCS provided by UNRWA were found in the annual health report published by the UNRWA [22], and [2] the global oral health status report [5].

### Data extraction strategy

The articles and reports that met the inclusion criteria were retained for data extraction. A data extraction chart collated and compared available OHCS inputs, outputs, and oral healthcare processes in WHO-EMR countries. Two reviewers developed this chart; one reviewer extracted the data to an electronic data form (Excel), and the second reviewer double-checked the extraction for the content. The extracted data was standardised based on the WHO standards for evaluating healthcare services the WHO framework for health system performance assessment within WHO-EMRO countries [23–25].

The final characteristics and data charting form were discussed by the research team and follow the WHO recommendations. The evaluation of oral health services profile and performance followed the WHO framework for health system performance assessment within WHO-EMR countries [23–25]. System performance is the extent to which health systems meet their goals [26]. Effective oral healthcare systems should achieve the main three cross-goals for the health system [23]: Health (Oral Health); Responsiveness; and Fairness in Financing.

The extraction table contains the following elements: Population size of the targeted country; Availability of OHCS; and Oral Health: a) Institutions providing OHCS, b) Personnel providing the care, c) Type of services: emergency, curative, prevention, treatment protocols, or referral system, d) Intervention and/or programmes for oral health prevention, e) Population size receiving the service? targeted age groups?, f) Inter-sectorial action: integration of oral healthcare in ongoing primary health programmes, g) Standard set of medical (dental) equipment and furniture, h) Information system for oral health practice and quality: information system for data collection, management, indicators and measurement of oral health outcome and quality of service, i) Staff capacity building: professional development and training programmes, j) Safety and accreditation programme, k) International health organisation support and contribution to OHCS, l) Responsiveness, m) Measurement to the respect of the oral health care system for persons (person’s dignity, autonomy, and confidentiality) and meeting client orientation (consumer satisfaction), n) Fair financing and financial risk protection, o),Health insurance coverage for oral health curative and prevention programmes, and p) Contribution of the national health budget to oral health care expenditure

### Risk of bias (quality) assessment

This review has not included studies assessing the effect size of a specific intervention. Thus, the methodological quality assessment and risk of bias assessment were not conducted as an essential part of the scoping reviews [27, 28].

### Strategy for data synthesis

The outcome of the review was presented using a descriptive-analytical method. A narrative summary of included studies and outcomes was presented in tables and discussed the findings' meanings and implications to the overall purpose of the review question. The tables included extracted data for the change in the incidence or indicators of oral health, responsiveness, and fairness in financing because of implementing OHCS.

## Results

One hundred thirty-seven studies met the review’s inclusion criteria in the databases searched. Of those, only 23 studies were retained for full-text review. Figure 1 shows that eight studies were excluded for the following reasons: not finding the full text of the record, the study not being conducted in one of the WHO-EMR countries, or the study design not evaluating the OHCS in these countries. In total, fifteen studies were included in the review for data extraction (Fig. 1). The included peer-reviewed studies were published between 1987 and 2016. In addition, two reports were identified from the grey literature: [1] the OHCS provided by UNRWA, found in the annual health report published by UNRWA [22], and [2] the global oral health status report [5].

The data related to oral and dental healthcare in the reviewed studies and grey literature is summarised in Tables 2, 3 and 4. While, the data related to oral and dental healthcare from the WHO global oral health report [5] was summarised in Table 5.

**Table 2:** The political and socio-demographic characteristics of countries included in the review

**Reference**	**Type of the Study**	**WHO-EMRO Country**	**Country Political Status**	**Demographic Characteristics**				**Fair Financing and Financial Risk Protection**	
				**Population Size (in Millions)**	**Population Growth Rate (%)**	**World Bank Country Classification + GNI/year (US Dollars)**	**Health Expenditure (%) of GNP or GDP/year**	**Health Insurance Coverage to OHC**	**National Health Budget Contribution to Oral Healthcare Expenditure (%)**
Abid, 2004 (Abid, 2004) [29]		Tunisia	Stable	7.9		Low and Middle Income		Basic dental care:	
Baghdadi, 2011 (Baghdadi, 2011) [30]	Review of OHCS	Kingdom of Saudi Arabia (KSA)	Stable	29		High-income		Basic dental care	
Basharat and Shaikh, 2016 (Basharat and Shaikh, 2016) [19]	Review of OHCS	Pakistan	Stable			Low and Middle Income			
Behbehani and Scheutz, 2004 (Behbehani and Scheutz, 2004) [20]	Review of OHCS	Kuwait	Stable	2.2 (in 2002)		High-income			
Behbehania and Shan, 2002 (Behbehani and Shah, 2002) [45]	survey	Kuwait	Stable			High-income			
Beiruti & van Palenstein Helderman, 2004 (Beiruti and van Palenstein Helderman, 2004) [16]	Review of OHCS	Syria	Stable	17 (in 2002)	2.60%	Low and Middle Income	3%		
Gallagher et al 2015 (Gallagher et al., 2015) [31]	Case study	Oman	Stable	3.8 (in 2013)	2% per year	High-income			
Guile and Shammary, 1987 (Guile and Shammary, 1987) [32]	Discussion paper	Saudi Arabian	Stable	8 (in 1986)	2.7% (in 1974)	High-income	2.81%		5% (In 1984).
Maatouk et al 2012 (Maatouk et al., 2012) [46]		Tunisia	Stable			Low and Middle Income			
Pakshir, 2004 (Pakshir, 2004) [33]	Review of OHCS	Iran	Stable	71		Low and Middle Income			
Peeran et al, 2014 (Peeran et al., 2014) [34]	Review of OHCS	Libya	Unstable	5.922 (in 2012)		Upper Middle Income	4%		
Shah et al, 2011 (Shah et al., 2011) [47]	Review of OHCS^a^	Pakistan	Stable	152		Low and Middle Income	0.70%		
Taani, 2004 (Taani, 2004) [15]	Review of OHCS	Jordan	Stable	5		Upper Middle Income			
Tahani et al., 2013 (Tahani et al., 2013) [35]	Observational, case study	Iran	Stable			Low and Middle Income			15.5% (in 2009)
UNRWA, 2023 (UNRWA, 2023) [22]	Annual Health Report	Palestine refugee camps: Jordan, West Bank, Gaza Strip, Syria and Lebanon	Unstable	5.9 (in 2022)	2% (1.3-2.9%)	Not Applicable	Not Applicable	Basic and preventive dental care	3.7% (in 2022)
Wissa and Zahran, 1988 (Wissa and Zahran, 1988) [36]	Observational study	Egypt	Stable	43.2 (in 1981)	29.7% in the rural areas	Low and Middle Income	2.90%		

^a^*OHCS* Oral Health Care Services

**Table 3:** Oral Health Services Profile in WHO EMRO Countries

**Reference**	**WHO-EMRO Country**	**Oral Health**								
		**Oral and dental diseases profile**	**GNP for oral health/year (US Dollars)**	**OHC included within the country’s national health plan: Yes/No**	**OHC providers**	**OHC Government/UNRWA contribution (%)**	**Population % receiving Government/UNRWA's service**	**Personnel providing OHC**		
								**No. Dentists**	**No. Auxiliary and administrative staff**	**Dentist to population ratio**
Abid, 2004 [29]	Tunisia	**Survey of 1994:** **Dental Caries experience and prevalence:** - 57% have dental caries in primary teeth - 2.1 DMFT in 6 year olds - 48% of 12 year olds had dental caries in permanent teeth - 58% of 15 year olds had dental caries in permanent teeth - 2 DMFT in 15 year olds. - Reduction in dental caries from 1981 to 1991 was significant (P<0.001) - The D-component was high - dental therapeutic index was very low - The DMF at age 12 was lower than WHO goals for the Year 2000 and of the Eastern Mediterranean Region countries. **Preiodontal disease:** - 60% in 12 year olds - 70% of 15 year olds **Malcoclusion prevalence:** - 38% in 12 year olds - 36% of 15 year olds - these were the highest percentage reported in the WHO_EMRO countries **Dental Fluorosis prevalence:** - Fluorosis was endemic in some regions - 18% among 6 year olds - 28% among 12 year olds - 31% among 15 years old **Toothbrushing practice was:** - 46% among 6 year olds - 86% 12 year olds - 92% 15 years old			- Ministry of Public health - private sector	- Ministry of Public health operates 321 dental clinics And Dental hospitals - 25% of public receive dental care from private sector.		1200 dentist (in 1993) distributed as follow: - 76% in the private, - 21% public, - 3% university sector. - There were an unequal distribution of oral health personnel, - the southern part of the country being particularly underserved. 1756 dentist (In 2004), distributed as follow: - 71% in private, - 21% in public, - 3% in university sector - 5% unemployed.	400 dental technician (in 1993) 600 dental technician (In 2004),	**In 1993:** **- In Tunisia:** - 1 dentist: 7872 inhabitants. - 1 dental clinic: 7872 inhabitants. **- In capital:** - 1 dentist: 2215 inhabitants. - 1 dental clinic: 2416 inhabitants. **In south Tunisia:** - 1 dentist: 120000 inhabitants. - 1 dental clinic: 41666 inhabitants. **In 2004:** **- In Tunisia:** - 1 dentist: 5770 inhabitants. - 1 dental clinic: 6091 inhabitants. - with certain regional disparities.
Baghdadi, 2011 [30]	Kingdom of Saudi Arabia (KSA)	In the Western province in Jeddah: - the caries prevalence was 85.5% for the deciduous dentition in 6 years old - the mean dmft scored 5.45 - the caries prevalence was 71.3% for the permanent dentition - the mean DMFT scored 4.9. - Similar caries severity and treatment needs were recorded in the school children of **the Southern, Central and Eastern Provinces of Saudi Arabia**. **In the capital city Riyadh:** - the caries prevalence was 74.8% in pre-school children - the mean dmft score of 6.1. - Similar high dmft scores were reported from **other regions of Saudi Arabia**. - children in the Arab Gulf countries are sharing the same nutritional problems, including - dental caries utilisation of oral health services among children and adolescents is low to medium and irregular. - Seeking relief from pain⁄toothache is the main reason given for visiting the dentist. - The routine of going for regular check-ups has not been established. - There is a general low interest in dental health care and preventive measures and this constitutes a major challenge to oral health.		Yes	- Ministry of Health, - universities, - Ministry of Defence and Aviation, - National Guard and - private dental centres.	- Oral healthcare, like all other health services, is mainly provided by the Ministry of Health. - The number of private hospitals is low and not all of them provide oral health care.		4,073 active dentists: - 1,581 work for the Ministry of Health, - 641 work for other government bodies - 1,851 work for the private sector		1:5,235
Basharat and Shaikh, 2016 [19]	Pakistan	**Dental caries prevalence:** -50–70% (in Karachi and Lahore). **Oral cancer** is among the highest in the world		Yes	Primary Health Care			Acute shortage of health workers, including dentists		1:10 850 - Low compared with the WHO recommended level of 1:7500
Behbehani and Scheutz, 2004 [20]	Kuwait	- The prevalence of dental caries in Kuwait is high and there is no indication of a decrease - Periodontal diseases affect the majority of the population. In 1988, 748 oral cancer cases were detected, and 75% of oral cancer cases were non-Kuwait.			- Ministry of Health - Faculty of Dentistry at Kuwait university.			- 745 dentists in the Ministry of Health - 168 dentists in private practice. - 26% of Dentists were specialists	- 121 laboratory technicians, - 775 dental nurses, - 81 oral hygenist, - 58 Xray technician.	1:2,867
Behbehania and Shan, 2002 [45]	Kuwait	**Between 1984 and 1985:** - Females were more often brushing their teeth at least once a day than males and non-Kuwaitis slightly more often than Kuwaitis - 39% had visited a dentist during the previous 12 months. -66% of subjects had a soft plaque, -45% had calculus -46% had intensive gingivitis -18% had advanced periodontitis **The caries experience:** -52% in the primary dentition (1-8 year olds) - 95% among the 60 to 64 year-olds - A distinct decline in caries experience during the 1980s was indicated (dmf/DMF was 8.4/0.4 in 1982 and 6.2/0.2 in primary and secondary dentition of 6-year-olds in 1993) - An increase in dental caries was indicated in 12-year-olds between 1982 and 1993 from 2.0 to 2.6. - The largest part of decayed permanent teeth was left untreated - extraction was twice more common as restorative treatment								
Beiruti & van Palenstein Helderman, 2004 [16]	Syria	**Caries experience:** **In 1985:** - 5.2 dmft and 77% caries prevalence in 5 years old group - 2 DMFT in 12 years old group - 7 DMFT in 25-34 years old group - 9.8 DMFT in 35-44 years old group **In** 1991**:** - 4.6 dmft and 74% caries prevalence in 5 years old group **In** 1998**:** - 2.3 DMFT in 12 years old group (D-componenet 80-90%) - 3.6 DMFT in 15 years old group (D-componenet 70-80%) - 11.2 DMFT in 35-44 years old group (M-component 40-50%) - high prevalence of untreated caries lessions in childhood. **Periodontal diseases:** - 6-15% of 15 years old and 35-44 years old Syrians were free of gingival bleeding, calculus and periodontal diseases. - 94% of 15 years old had dental plaque accumulated - 3-11% of 35-44 years old Syrian had sever periodontal diseases. **Fluoride level in water and Fluorosis prevalence:** - Water fluoride concentration 0.8-1.9 ppm F. - 78% of 13-15 years old children in Palmyra had dental fluorosis.		Yes	- Ministry of Health - Ministry of Education providing School Oral Health Programme - Four dental faculties - Private sector - Military Medical services		8% of the population benefit from the Ministry of health’s oral health care.	14,610 (In 2002): 1800 were working in the Ministry of Health	In 2002: - 750 dental hygienists - 4000 dental technicians. In 1990: - 40 dental hygienists and nurses were working in the Ministry of Health	1:1172 (In 2002)
Gallagher et al 2015 [31]	Oman	- 5 DMFT and 85% caries prevalence in 6 years old group **In 1991:** - 2.5 DMFT and 85% caries prevalence in 12 years old group **In 2006:** - 1.3 DMFT and 51% caries prevalence in 12 years old group		Yes	- Ministry of Health - Military ser vices			726 (in 2011) 654 (in 2010): - 24% of the dentist was Omani - 53% of dentists were working in the Ministry of Health, - 68% were working in military services - 1% were in the private sector		- 2.3 dentists per 10 000 population - lower than the Gulf Cooperation Council (GCC) average (3.2 per 10 000)
Guile and Shammary, 1987 [32]	Saudi Arabian	**Dental Caries experience:** DMFT 2.7 (among Riyadh 13 years old children examined in Pilot study conducted in 1981)		Yes	- Ministry of Health, - National Guard - Ministry of Defence - Military services (provides dental care to civilians in areas of dental personnel shortage) - Private sector	70%	100%	280 (In 1980) 850 dentists (In 1986) of which 550 dentists working in the Ministry of Health	Ministry of Health trained technicians, dental assistants and dental therapists and added them to the school-based dental care system	1:28,000 (In 1980) 1:9,411 (In 1986)
Maatouk et al 2012 [46]	Tunisia	**Survey of 2003:** **Dental Caries experience and prevalence:** - 56.3% of 6 year olds have dental caries in primary teeth - 49.1% of 12 year olds had dental caries in permanent teeth. - 1.4 DMFT in 12 year olds, with: - a very high D component (1.33) - a low F component (0.03) - a great insufficiency in treatment. - more than 73% of 35 to 44 years olds - 5.6 DMFT in 35 to 44 years olds, constitutes: - mainly of D component (3.0) - a low therapeutic index (0.45) **Other oral health indicators:** - poor oral hygiene was common among 15-year-old schoolchildren and periodontal troubles (especially calculus) constituted a serious problem in 38% of them. - more than 73% of 35 to 44 years olds suffer from periodontal disease - malocclusion was more frequent in schoolchildren (40.7%) in Tunisia than in other countries of the East Mediterranean Region (EMRO) - dental fluorosis was endemic in some regions of the country (40.7%) as in some other African countries **A survey in 2008, involved 816 adults aged 65 years and over:** - more than half of the subjects had dental caries while - 80% had periodontal problems and 32% were totally edentulous.								
Pakshir, 2004 [33]	Iran	**Dental caries status from Nationwide survey in 1990-1992:** - 0.3 DMFT and 88.6% were caries-free in 6 years old group - 2.4 DMFT and 68.7% caries prevalence in 12 years old group - 5 DMFT and 87.3% caries prevalence in 15-19 years old group - 11.3 DMFT and 98.8% caries prevalence in 35-44 years old group **Dental caries status from Nationwide survey in 1995:** - 2.02 DMFT and 17% were caries-free in 12 years old group **Dental caries status from Nationwide survey in 1998-1999:** - 1.8 dmft and 46.8% caries prevalence in 3 years old group - 4.8 dmft, 0.2 DMFT and 85.9% caries prevalence in - 6 years old group - 3.4 dmft, 0.9 DMFT and 84% caries prevalence in 9 years old group 1.5 DMFT and 67.2% caries prevalence in 12 years old group **Dental caries status from Nationwide survey in 2001-2002:** - 4.1 DMFT and 87.3% caries prevalence in 15-19 years old group - 14.8 DMFT and 98.8% caries prevalence in 35-44 years old group **Periodontal diseases from Nationwide survey in 2001-2002:** - 70% had bleeding or calculus in 15-19 years old group - 50% had severe periodontitis requiring extensive periodontal treatment in 35-44 years old group			Public and private sectors		- 70% of oral health services provided by government in rural areas - 80% of oral health services provided by private sectors in cities:	**13,000 dentists:** - 10% working in OHC in the public sector - 79% in private sector **1200 dentist specialists:** working in either universities or in private	- 2000 Oral Hygienists provide dental care services in rural areas and afterwards, reduced to 650 - 35000 Behvarzes in rural areas provide oral education	1 dentist: 5,500 population
Peeran et al, 2014 [34]	Libya				Public and private sector					six dentists per 10,000 persons (In 2012)
Shah et al, 2011 [47]	Pakistan	**Caries prevalence:** - 50% among 12–15 years - 98.5% of caries were untreated (In 1992) - high prevalence among adults aged 65 and over **Periodontal Health Status:** - < 28% of 12-year-old children (12-year-old) have healthy gingiva - 20%t of women have bleeding gingiva - 34% have dental calculus - 17% of women (18–34-year-old) have advanced periodontal diseases, - 93% of 65-year olds have some form of gingivitis or periodontitis. **Oral cancer** is the second most common cancer in Pakistani	No record of annual oral healthcare expenditures is available	Yes	- Pakistan Medical and Dental Council (PMDC) - Federal Ministry of Health		55 %	8,169 (In 2008)	Two dental hygiene diploma programs, each graduated 15–20 dental hygienists/year	Insufficient supply and distribution of oral health professional
Taani, 2004 [15]	Jordan	**Dental Caries experience:** In the years 1984, 1990 and 2002: -DMFT was 4, 5 and 2.5 respectively in among 12 years old children D-component accounted for 89-91% of DMFT. **Other oral disorders:** in 2003: -92% of 13-15 y children in North Jordan had mal occlusion. - 79% of trauma was a crown fracture. - The prevalence of periodontal diseases is the highest among 50-60-year-olds. - 19% and 11% of 50 and 60 years olds had deep periodontal pockets - 1/3 of children (35% in public schools and 57% in Private schools) and more than half of adults brushed their teeth regularly - Public school children had higher DMFT and DMFS than children in private schools - plaque and gingivitis were high at all socioeconomic levels - plaque score was significantly higher in very poor and poor groups.								
Tahani et al., 2013 [35]	Iran	**Caries experience:** -2 dmft among 3-year old (In 2007). In 2013: -5 dmft, among 6 years of age (with decayed teeth component equals 95%) -1.86 DMFT at the age of 12. -11 (± 6.4) DMFT among adults		Yes		- Ministry of Health - Medical Education and private sector				
UNRWA, 2023 [22]	Palestine refugee (PR) camps in five fields of operation (Jordan, West Bank, Gaza Strip, Syria and Lebanon	In 2016: PREVALENCE of Dental Caries (DMFT/S>0), DMFS, DS/DMFS% and FS/DMFS for 11-13 years schoolchildren: Overall: 72.8%, Jordan: 68.4 % - 3.23 - 76.9% - 17.0% Lebanon: 73.6% - 3.78 - 79.2% - 20.3% Syria: 45.9% - 4.22 - 80.0% - 18.0% Gaza: 70.7% - 2.66 - 82.9% - 12.7% West Bank: 79.7% - 5.88 - 85.4% - 9.2%		Yes	UNRWA	5,412,923 US Dollars		116		1:50772.8
Wissa and Zahran, 1988 [36]	Egypt				Government			100 dentists working in 60 rural dental care centres

**Table 4:** Oral health related programmes and responsiveness indicators in the included studies

**Reference**	**WHO-EMRO Country**	**Oral Health**									**OHC Responsiveness**
		**OHC programmes**									
		**OHC promotion and prevention**	**Basic OHC services**	**OHC-related research**	**OHC Surveillance**	**Multisectorial networking**	**OHC stratigic plan**	**Dental equipment and furniture availability**	**Staff capacity building and training**	**Safety and accreditation programme**	**Indicators (person’s dignity, autonomy, confidentiality and/or satisfaction)**
Abid, 2004 [29]	Tunisia	school oral health established by the Ministry of Health in 1992, provide: - screening by dentists for elementary, secondary and high-level students - refer them to dental care in hospitals	filling and tooth extraction		school oral health surveys carried out in 1981, 1984, 1988 and 1994 using WHO oral health examination criteria.					one dental school graduate dentists (66 years study) and a dental technician (3 years of study).	
Baghdadi, 2011 [30]	Kingdom of Saudi Arabia (KSA)	- a national campaign against tooth decay among children. - the immediate aim in the next 5 years is to treat one million children suffering from tooth decay. – The campaign aims to reduce the rate from the current 90% to 75%. - Other aims of the campaign are to raise awareness of oral health, emphasising the culture of prevention of tooth decay, encouraging sound habits of oral care, including cleaning after every meal, eating a healthy diet, reducing sugars and periodically visiting the dentist. - The campaign launched a website and asked for volunteers’ participation and registration.	- The traditional restorative approach to managing dental caries has been deeply integrated into the legislative and remuneration systems, - the dental school curricula, and public knowledge, mergency fillings and extractions are normally inclueded in standard health plans. - Most of other dental treatments, including prevention, are usually not included.	- Fluoride mapping has shown that the majority of the Saudi regions have lower fluoride llevelsthan recommended levels. - Also, brushing their teeth daily is not one of the firm habits in Saudi children; about 25% of children rarely or never brush their teeth and few used toothpaste before 3-years of age39. - more than 40% of the Saudi children were not brushing their teeth.	Available				- 10 private dental colleges, - there are 393 dental graduates each year		
Basharat and Shaikh, 2016 [19]	Pakistan		Curative dental services are available at the primary health care level in Pakistan in only a few places and cannot deal with the burden of oral cancer and tooth decay								
Behbehani and Scheutz, 2004 [20]	Kuwait										
Behbehania and Shan, 2002 [45]	Kuwait				national oral health surveys were conducted among 5- to 16-year-old children in 1982 and among 4-, 6-, 12- and 15-year-olds in 1993						
Beiruti & van Palenstein Helderman, 2004 [16]	Syria	Ministry of health provides: - dental education for pregnant women by nurses. - Provision of Fluoride tablets for infants. -School Oral health education and promotion provided by dentist, nurses, assistants and trained school teachers to 50% of student at grade 1 and 6 in schools. In 1998 Oriented school Health curriculum was adopted to improve oral health student for students and their paprents: - 12% of students at the 2nd and 6th grade in schools received Fluoride mouth rinse, programme stopped because of a lack of teacher compliance and administrative obstacles. - 5% of the 2nd and 5th grade children receiving curartive denatal care in 105 school dental clinics (including 210 dentist and 115 dental hygienists), services are restoration (ART), fissure sealant, periodontal treatment and extraction.	- Ministry of health providing curative services (restoration, scaling, extraction and emergency care). - in 1990, 476 Maternal and Child Health centres provided dental care and 1,800 dentists worked in these clinics. Average of 3 dentist per clinic. 40 dental hygienist were employed in these centres. - Private sector provides specialist treatment and almost exclusively located in cities and towns. - Military Medical services provides to dental care for employees in Interior Ministry and their families. - Some industries have insurance system and refer their employees to private clinics.							Four dental faculties: Damascus, Aleppo, Hama and Lattakia. Damascus, Aleppo, and Hama operating intermediate dental institutes graduating yearly 300 dental technicians and 50 dental hygienist.	
Gallagher et al 2015 [31]	Oman	- introduction of water fluoridation in the Muscat area, together with school oral health prevention programmes involving fissure sealants, tooth brushing and the application of fluoride - the Ministry of Health has had a number of ongoing community initiatives for oral health in schools, to contribute to recent promising improvements in oral health amongst the 12-year-olds in the Sultanate									
Guile and Shammary, 1987 [32]	Saudi Arabian	- Fluoride applications to school-age children and provided in Clinics. - Water Fluoridation is provided to areas that benefit from the centralised piped water supply.	Curative services								
Maatouk et al 2012 [46]	Tunisia	- the National Oral Health Programme was founded in 1991 by the Public Health Ministry. - It provides preventive measures			4 national oral health surveys the last one was in 2003.						
Pakshir, 2004 [33]	Iran	First level of PHC, Behvarzes are health workers providing primary health care (PHC) services including: - oral education in rural areas (health house level of PHC), - periodic examination of teeth, - referral to a higher level - follow-up of outcome - supervising sodium Fluoride mouth rinse.	At PHC second level Oral Hygienists and dentists in rural areas provide: - simple fillings, scaling, extraction and fluoride therapy - supervise the activities of Behvarzes in the Health house. At PHC level 3, dentists and dental nurses and technicians manage of treatment of dental and oral diseases as tertiary prevention. At PHC level 4, advanced treatment is offered by specialists in University Health Centres in the Cities.				- The Oral health department at the Ministry of Health and Medical Research is responsible for policy-making and planning for oral health care at the National Level. - Strategy: promoting community oral health through increasing of public awareness and qualitative and quantitative improvement of oral health care. - It published posters, books, films and prepared and distribute ID cards. - National School health programmes targeted children 6-12 years old and provide health education, supervised tooth brushing and weekly mouth rinsing (0.2% sodium fluoride mouth rinse) and providing extraction, tooth restorations and fluoride therapy.				
Peeran et al, 2014 [34]	Libya	Government expenditure in respect to oral health follows this outdated model and targets on the diagnosis of and treatment for oral and dental diseases rather than on oral health prevention programs.	- The Health Ministry provides dental health services to people of all ages through the public dental clinics with dental health services generally spread throughout the cities. - The main treatments are minor oral surgery, tooth scaling, and restorations with very little development of preventive services. - Public dental clinics deliver simple oral examinations, scaling, tooth extractions, and dental fillings.	Extremely Low							
Shah et al, 2011 [47]	Pakistan	Non-exist	- Pakistani citizens, especially rural residents, lack access to adequate, affordable, and organized oral health services. - 90% of all the treatment provided in public dental clinics is tooth extraction. - Oral examination, scaling and prophylaxis count for <3% of the services provided in public dental clinics				- The mission of the dental hygiene profession in Pakistan is to promote a high standard of dental hygiene practice. - a dental hygienist uses preventive, educational, and therapeutic methods to promote oral health and prevent and control oral diseases.	40% of the dental equipment at rural health centres (RHCs), is non-functional and dental materials, instruments and drugs are excluded from the RHCs’ essential supply list. - The public healthcare sector provides preventive and therapeutic services through rural RHCs	The dental hygiene profession’s vision is to attract competently members who can collaborate with other health professionals to improve general and oral health and quality of life for all. Given this vision, the profession foresees the development of strong academic programs for dental hygienists, effective rules and regulations that govern practice, and mechanisms to ensure quality practice over the dental hygienist’s lifetime		
Taani, 2004 [15]	Jordan				National Epidemiological Studies included oral health examination in 1984 and 1990.						
Tahani et al., 2013 [35]	Iran	Water fluoridation	Programs now available for disadvantaged groups are mostly those focusing on the provision of dental care through primary health care (PHC) by dentists or dental hygienists	Oral health faces shortcomings in the management of information and analysis of available data to use these data for bureaucratic purposes.							All health care professionals, including dentists, are responsible for the damage they cause during their medical practice, although there is no specific legislation for malpractice and they are examined under the general role of law.
UNRWA	Palestine refugee (PR) camps in five fields of operation (Jordan, West Bank, Gaza Strip, Syria and Lebanon	UNRWA's health services continue to prioritize preventive oral health components by raising awareness of the importance of preventative oral health during routine MCH care and providing preventive dental care for newly registered NCD patients. This includes dental screenings for women during their first preconception care visit and all pregnant women, as well as comprehensive oral health assessments for children at the age of one and two, and application of fluoride varnish starting from one year of age and then twice a year until they reach five years old. The Oral Health Programme's staff conducts regular oral health assessments for preschool children and dental screenings for new school entrants, second, fourth, and seventh-grade students, and applied pit and fissure sealant for first and seventh-grade students. Additionally, oral hygiene education continues for all school students across all fields to prevent oral health problems.	Among the 130 dental clinics providing these services, 119 are located within the Agency's primary HCs, while the remaining 11 are mobile dental clinics.	the Agency conducts operational research on oral health in line with World Health Organization (WHO) guidelines and UNRWA's needs to track disease trends and improve the overall oral health status	The Oral Health Programme's staff conducts regular oral health assessments for preschool children and dental screenings for new school entrants, second, fourth, and seventh-grade students, and applied pit and fissure sealant for first and seventh-grade students. Additionally, oral hygiene education continues for all school students across all fields to prevent oral health problems. An assessment of the Oral Health Programme’s staff workload, needs, productivity, and efficiency is conducted in all five fields annually. The Health Programme uses a standardized counting unit to measure the technical workload of the staff, and this assessment is conducted periodically as part of the performance evaluation process. This assessment is also used to identify staffing requirements and the need for reorganizing oral health services. Furthermore, UNRWA conducted an additional assessment in collaboration with WHO/EMRO to evaluate the impact of oral preventive services and to identify improvement opportunities.		The primary objectives of the oral health services are (i) to prevent, detect, and manage dental and periodontal disorders, with particular attention paid to at-risk groups; (ii) to promote oral hygiene through active screening, management of vulnerable populations, and targeted health education activities promoting the use of fluoride toothpaste; (iii) to provide an equitable service and expand public health interventions to address oral health determinants; and (iv) to improve ongoing collection, analysis, and interpretation of health data for planning, implementation, monitoring, and evaluation.				
Wissa and Zahran, 1988 [36]	Egypt	No preventive programme included in rural health centres programmes	- extraction of primary teeth, scaling, prophylaxis: - temporary dressing and prescribing antibiotics for acute cases - conservative treatments (dental filling). - services are free for everyone.					- Complete dental equipment (usually has a conventional motor engine, sometimes a foot engine was found in addition to the motor engine) - available in 31% of rural health centres have dental facilities, which equals 612 centres out of 2468 centre

**Table 5:** Oral care services profile in the WHO-EMRO countries (World Health Organization, 2022)

**Countries income based into four strata based on DAC List**	**Country**			**Oral disease burden**			
		**Total population (at the year 2020)**	**Per capita current health expenditure in International Purchasing Power Parity (PPP) dollar US$**	**Prevalence of untreated caries of deciduous teeth in children 1-9 years (%)**	**Prevalence of untreated caries of permanent teeth in people 5+ years (%)**	**Prevalence of severe periodontal disease in people 15+ years (%)**	**Lip and oral cavity cancer, all ages: Incidence rate (per 100 000 population)**
High	Bahrain	1 477 000	1881	42.8	34.3	20.4	1.6
High	Kuwait	4 360 000	2861	43.5	33.6	19.1	1.6
High	Oman	4 543 000	1161	45.5	34.3	15.6	1.9
High	Qatar	2 760 000	2737	46.0	33.6	18.2	2.2
High	Saudi Arabia	35 997 000	2790	53.2	38.8	10.8	1.7
High	United Arab Emirates	9 287 000	2996	50.8	33.8	1.1	1.5
Upper-Middle	Iraq	42 557 000	483	43.9	35.1	13.3	1.1
Upper-Middle	Jordan	10 929 000	798	42.8	38.5	13.8	1.3
Upper-Middle	Lebanon	5 663 000	1298	43.8	34.9	16.7	1.1
Upper-Middle	Libya	6 654 000		44.1	36.0	15.3	1.1
Lower-Middle	Egypt	107 465 000	582	44.1	30.4	14.2	1.6
Lower-Middle	Iran	87 290 000	868	46.7	33.6	18.0	1.3
Lower-Middle	Morocco	36 689 000	425	44.4	36.0	19.7	1.8
Lower-Middle	Pakistan	227 197 000	166	45.7	27.0	24.1	10.1
Lower-Middle	Palestine: Occupied Palestinian territory	No data available					
Lower-Middle	Tunisia	12 162 000	789	44.1	34.8	17.3	1.7
Lower-Middle	Syrian Arab Republic	20 773 000		39.3	36.9	14.2	1.2
Least Developed	Afghanistan	38 972 000	286	45.8	36.8	9.4	4.6
Least Developed	Djibouti	1 090 000	104	39.7	31.1	24.3	1.8
Least Developed	Somalia	16 537 000		43.0	33.7	16.2	2.0
Least Developed	Sudan	44 440 000	205	42.1	35.5	10.9	2.1
Least Developed	Yemen	32 284 000		45.0	36.8	10.3	1.7
Unclassified	UNRWA OR Refugee	No data available					

**Countries income based into four strata based on DAC List**	**Country**	**Risk factors for oral diseases**	**Economic impact related to treatment and prevention of oral diseases**						
		**Per capita availability of sugar (g/day)**	**Prevalence of current tobacco use, 15+ years (%)**	**Per capita alcohol consumption, 15+ years (litres of pure alcohol/year)**	**Total expenditure on dental healthcare in million (US$)**	**Per capita expenditure on dental healthcare (US$)**	**Total productivity losses due to 5 oral diseases in million (US$)**	**Affordability of fluoride toothpaste**	**Number of labour days needed to buy annual supply of fluoride toothpaste per person**
High	Bahrain		15.2	1.1	62	41	148		
High	Kuwait	94.6	17.9	0.0	218	46	374		
High	Oman	56.6	7.9	0.9	133		232		
High	Qatar		12.0	1.5	229	83	495		
High	Saudi Arabia	82.9	14.2	0.0	1 252	37	2 016		
High	United Arab Emirates	94.4		3.9	699	65	1 256		
Upper-Middle	Iraq	47.4	18.8	0.4	0	0	581		
Upper-Middle	Jordan	82.0	34.6	0.5	75	7	120	unaffordable	2.1
Upper-Middle	Lebanon	108.7	38.4	1.5	105	17	183	affordable	0.5
Upper-Middle	Libya	77,1		0.0	61	9	130		
Lower-Middle	Egypt	70.9	24.4	0.1	329	3	826	unaffordable	1.1
Lower-Middle	Iran	74.6	14.0	1.0	3 507	42	1 799		
Lower-Middle	Morocco	91.8	14.9	0.5	82	2	452		
Lower-Middle	Pakistan	57.7	20.8	0.3	49	0	617		
Lower-Middle	Palestine: Occupied Palestinian territory								
Lower-Middle	Tunisia	90.6	25.0	2.0	61	5	143		
Lower-Middle	Syrian Arab Republic	57.3		0.2	28	2	48		
Least Developed	Afghanistan	46.8	23.8	0.0	17	1	37		
Least Developed	Djibouti	112.8		0.4	1	1	7		
Least Developed	Somalia				3	0	8		
Least Developed	Sudan	77.3			46	1	74		
Least Developed	Yemen	66.7	20.5	0.0	10	0	46		
Unclassified	UNRWA OR Refugee								

**Countries income based into four strata based on DAC List**	**Country**	**National health system responses**						
		**Policies, measures and resources (2021)**	**Oral health workforce (Per 10 000 population)**					
		**Implementation of tax on sugar-sweetened beverages (SSB)**	**Existence of a national oral health policy/strategy/action plan (operational/ drafting stage)**	**Presence of dedicated staff for oral health working on NCDs at the MoH**	**Noma recognized as a national public health problem**	**Dental assistants and therapists**	**Dental prosthetic technicians**	**Dentists**
High	Bahrain	yes	yes	yes	no		0.5	1.0
High	Kuwait	no	yes	yes	no			6.7
High	Oman	yes	yes	yes	no	0.6		3.0
High	Qatar	no	yes	yes	no	2.8	0.4	6.1
High	Saudi Arabia	yes	yes	yes	no		0.9	5.0
High	United Arab Emirates	yes	yes	yes	no			6.5
Upper-Middle	Iraq	no	yes	yes	no	0.2	0.3	2.6
Upper-Middle	Jordan	no	yes	yes	no			7.1
Upper-Middle	Lebanon	no	no		no			10.2
Upper-Middle	Libya	no	no	yes	no			8.8
Lower-Middle	Egypt	no	no	yes	no		0.2	2.0
Lower-Middle	Iran	yes	yes	yes	no			4.5
Lower-Middle	Morocco	yes	yes	yes	no			1.4
Lower-Middle	Pakistan	yes	no	no	no	0.2		1.2
Lower-Middle	Palestine: Occupied Palestinian territory	No data available						
Lower-Middle	Tunisia	yes	yes	yes				3.1
Lower-Middle	Syrian Arab Republic	no	yes	no	no			7.2
Least Developed	Afghanistan	yes	no	yes	no	0.2	0.1	0.7
Least Developed	Djibouti	no	no	no	no			0.2
Least Developed	Somalia	no	no	no	no			
Least Developed	Sudan	no	no	no	no			2.1
Least Developed	Yemen		no	no	no			0.2
Unclassified	UNRWA OR Refugee	No data available						

**Countries income based into four strata based on DAC List**	**Country**	**National health system responses**							
		**Availability of procedures for detecting, managing and treating oral diseases in the primary care facilities in the public health sector**	**Oral health interventions as part of health benefit packages**						
		**Oral health screening for early detection of oral diseases**	**Urgent treatment for providing emergency oral care & pain relief**	**Basic restorative dental procedures to treat existing dental decay**	**Coverage of the largest government health financing scheme(% of the population)**	**Routine and preventive oral health care**	**Essential curative oral health care**	**Advanced curative oral health care**	**Rehabilitation oral health care**
High	Bahrain	available	available	available					
High	Kuwait	available	available	available		yes	yes	yes	yes
High	Oman	available	available	available	99	yes	yes	yes	yes
High	Qatar	available	available	available					
High	Saudi Arabia	available	available	available					
High	United Arab Emirates	available	available	available	30	yes	yes	yes	yes
Upper-Middle	Iraq	available	available	available					
Upper-Middle	Jordan	unavailable	available	available					
Upper-Middle	Lebanon	unavailable	available	unavailable	50	no	no	no	no
Upper-Middle	Libya	available	available	available					
Lower-Middle	Egypt								
Lower-Middle	Iran	available	available	available	95	yes	yes	yes	no
Lower-Middle	Morocco	available	available	available	60	yes	no	no	no
Lower-Middle	Pakistan	available	available	available					
Lower-Middle	Palestine: Occupied Palestinian territory								
Lower-Middle	Tunisia	available	available	available	72	yes	yes	yes	yes
Lower-Middle	Syrian Arab Republic	available	available	available	99	yes	yes	yes	yes
Least Developed	Afghanistan	available	available	available					
Least Developed	Djibouti	available	available	available					
Least Developed	Somalia	unavailable	unavailable	unavailable	41	yes	no	no	no
Least Developed	Sudan	unavailable	available	unavailable					
Least Developed	Yemen								
Unclassified	UNRWA OR Refugee								

### Demographic characteristics of the included populations

Based on the list of Development Assistance Committee (DAC) in the United Nations [37], the included studies reviewing the OHCS in three high-income countries (Saudi Arabia, Kuwait, Oman), two upper-middle-income countries (Libya, Jordan) and five LMICs (Iran, Egypt, Syria, Pakistan, Tunisia). The information about OHCS provided by UNRWA to the Palestinian refugees living in the refugee camps located in the Palestinian Territories (Gaza Strip and West Bank), Jordan, Syria, and Lebanon was extracted from the most recent UNRWA Health Department Annual Report for the year 2022 [22].

Table 2 summarises the political and socio-demographic characteristics of the countries in the included studies. Most countries had stable political statuses when the studies were conducted, except for Libya [34] and Palestinian refugees’ camps [22]. Among the included studies, Pakistan had the highest populous size (~ 152 million), whereas Kuwait was the lowest (~ 2.2 million) [20]. Population growth rates ranged between 2% and 2.7%. In 1988, the reported growth rate in the rural areas of Egypt (29.7%) was the highest in the WHO-EMR and the world [36].

In 2022, Pakistan's population doubled (227.2 million) and remained the highest in the region. Meanwhile, Djibouti (1.1 million) accounted for the lowest population size, as shown in Table 4.

### Healthcare and oral healthcare services

The assessment of the performance of OHCS in this review followed the WHO framework for health system performance assessment and health services research tools [1, 23–25, 38].

In included countries, governmental MOHs are the dominant provider of these services. UNRWA is the main provider of OHCS for Palestine refugee camps in five fields of operation (Jordan, West Bank, Gaza Strip, Syria, and Lebanon) [13] (Table 3).

The contribution percentage of Gross Domestic Product to Health Expenditure/year was the lowest in Pakistan (0.7%) and the highest in Libya (4%). Oral healthcare services accounted for 5%, 15.5%, and 3.7% of the national health budgets in Saudi Arabia (1984), Iran (2009) and UNRWA (2022) respectively (Table 2). Table 5 shows that in 2021, the contribution of per capita National health expenditure to per capita oral health expenditure was the highest in Iran (4.8%). In the high-income countries, it ranges between 3% in Qatar and 0% in Oman, while for all the rest of the countries, it is less than 1.3%.

The information regarding health insurance coverage for OHCS is rare; only Tunisia, Saudi Arabia, and UNRWA illustrated that health insurance covers essential treatments (fillings and extractions) and preventative dental care where available.

The oral health profile, services, and workforce are detailed in Tables 3 and 4. Syria, Oman, Iran, Saudi Arabia, Pakistan, Tunisia, and Kuwait included OHCS within the country’s national health plan. No study provided any information about gross national product allocation for oral health per year.

In some countries, the private sector, the Ministry of Education (for school oral health programmes), the Ministry of Defence (military medical services) and schools of dentistry in the local universities contribute to OHCS. The proportion of the population receiving OHCS from government MOH was reported only in Syria (8%), Pakistan (55%), Iran (70% by government in rural areas and 80% by private sectors in cities) and Saudi Arabia (100%), as shown in Table 3.

### Oral and dental health status

Table 3 illustrates that dental caries and oral diseases were highly prevalent in the countries included and significantly higher in poor groups. Pakistan had the second-highest oral cancer prevalence in the world [19]. Dental caries prevalence and experience (decay, missing and filled deciduous teeth (dmft) and Decayed, Missing, and Filled Permanent Teeth (DMFT)) was high among children and adults in all the reviewed studies and increased over the years. For example, dental caries in Iran were highly prevalent in all age groups (up to 98.8%). Moreover, the prevalence of dental caries and periodontitis was higher among older age groups [33, 35]. A large part of decayed primary and permanent teeth was left untreated in Syria [16]. The prevalence of gingivitis and periodontal disease is also extraordinarily high, with high plaque accumulation as it reached 94% among 15-year-old children in Syria. Tunisia had the highest prevalence of malocclusion compared to other EMR countries [29, 39]. Poor oral health and toothbrushing practices were common among most of the included populations, except in Tunisia, which reported good toothbrushing practices among its population [29] despite the high prevalence of dental caries.

Table 5 shows that, most recently, Saudi Arabia had the highest level of untreated deciduous and permanent decayed teeth (53.2% and 38.8%, respectively) in the region despite it being a high-income country. Generally, sugar intake is extremely high among the population of the EMR (46.8 - 112.8 g/day).

### Workforce in oral healthcare

Dentist to population ratio varies widely between countries; a shortage in this ratio was detected in Pakistan and southern Tunisia (1 dentist: 120,000 inhabitants). The availability of dental auxiliary and administrative staff was deficient in the studies included, mainly in Ministries of Health. Libya, Egypt, Oman, Bangladesh and UNRWA provided no information (Table 3).

Recent figures show countries where the dentists to population ratio is less than 2:10,000 are Yemen, Djibouti, Afghanistan, Bahrain, Pakistan, and Morocco. Most countries do not have a national oral health policy or action plan. Thus, oral health has not yet been incorporated into the NCD programmes at the Ministries of Health (Table 5).

### Oral healthcare curative and preventive services (Table 4)

The OHCS programmes and responsiveness information are presented in Table 4. Water fluoridation for the prevention of dental caries is applied in Saudi Arabia, Iran, and Oman. Most of the included studies' oral health prevention programmes varied between screening and applying fissure sealant and fluoride modalities (e.g., fluoride mouth rinse, toothbrushing with fluoride toothpaste and fluoride varnish).

Oral healthcare-related research and surveillance are rare. In addition, there are remarkable shortcomings in the management of information and analysis of available data to use these data for bureaucratic purposes. Only UNRWA illustrated that an annual assessment of the oral health programme’s staff workload, needs, productivity, and efficiency is conducted in all five fields. Otherwise, existing dental care prevention programmes were not assessed for their effectiveness. Finally, all health planners in the included studies agree on shifting from curative to preventive oral health strategies.

The countries applying tax strategies on sugary diets are Bahrain, Afghanistan, Oman, Pakistan, Iran, Saudi Arabia, Tunisia, Morocco, and the United Arab Emirates (Table 5). Oral health screening is unavailable in Jordan, Sudan, Lebanon, and Somalia. The latter three countries do not provide basic restorative dental procedures to treat existing dental decay. In addition to that, Somalia does not offer urgent treatment for emergency oral care or pain relief. Countries that included oral health interventions in governmental public Health Benefit Packages (routine and preventive oral healthcare, essential curative oral healthcare, advanced curative oral/dental care, and rehabilitative oral healthcare) are Oman, Tunisia, Iran, the United Arab Emirates, Kuwait, and Syria (Table 5).

## Discussion

This review found that few studies were published for evaluations of the OHCS in the countries of the WHO-EMR. Most recently, the WHO Global Oral Health Status Report [5] summarised OHCS profiles in these countries.

As mentioned, the included studies were published between 1987 and 2016. This was when most of the EMR countries were in stable political situations. Even so, dental caries and other oral health diseases were highly prevalent during that period. In addition, most of the included studies reported the OHCS's failure to meet the served population's oral healthcare needs. The enormous increases in political unrest in the WHO-EMR since 2016, and specifically in the most recent decade, undoubtedly worsened oral health status among the region's population. This may explain why more than half the children (1-9 years) had untreated caries in the deciduous teeth [5]. Nonetheless, the WHO report lacked information about Palestinian refugees’ oral health needs and services in the region. Moreover, the availability of dental public health education and capacity building for dental public health in the higher education sector were rare.

The governments’ neglect of OHCS provision is a well-known global issue [4]. The review results show that the contribution of national health expenditure to OHCS was extremely low (<5%), even in high-income countries. This explains why the information on health insurance coverage for the OHCS is rare. Moreover, any contribution of health insurance to OHCS was limited to covering the basic treatments (fillings and extractions) and prevention of dental care if it existed, as in Tunisia and Saudi Arabia [29, 30]. Iran operated a unique OHCS system, that depended on health workers in early detection and preventive oral health care, mainly in rural areas [33, 35]. Nonetheless, dental caries is still high among its population.

As in most countries worldwide, the provision of healthcare, including OHCS, is the responsibility of MOHs in the WHO-EMR [1, 23], with UNRWA the leading provider of healthcare services for Palestinian refugee camps situated in five fields of operation (Jordan, West Bank, Gaza Strip, Syria and Lebanon) [13]. Based on that, the WHO raised the necessity of elevating governmental support and consideration of the OHCS in the national and regional healthcare strategy [40].

Most of the included studies recommended this initiative and considered it part of future strategies to empower OHCS nationally. Additionally, other governmental and non-governmental health stakeholders may participate in the provision of OHCS, such as the private sector, the Ministry of Education (mainly for school oral health programmes), the Ministry of Defence (Military Medical Services) and Schools of Dentistry in the local universities. This would be an excellent opportunity to overcome the services shortage that the ministries of health are facing.

The assessment of the performance of OHCS in this review followed the WHO framework for health system performance assessment and health services research tools [1, 23–25, 38].

This review considered assessing the health workforce, administration, and supply of health services, OHCS quality and performance evaluation, and financial and technological support. High- and upper-middle-income countries (Saudi Arabia, Qatar, United Arab Emirates, Kuwait, Jordan, Libya, and Lebanon), in addition to Syria, reported dentist-to-population ratios of more than or equal to five dentists per 10,000 population. This is better than the worldwide dental workforce profile, which shows that over 68% of WHO Member States had less than five dentists per 10,000 population, and 37% had less than 1 [40]. A persistent shortage of dental assistants and technicians is a substantial issue in the WHO-EMR.

It is unclear whether water fluoridation interventions cover all populations in Saudi Arabia, Iran, and Oman [31, 32, 35]. However, water fluoridation is a highly recommended intervention to reduce dental caries among the whole population [41], combined with school-supervised toothpaste brushing with fluoridated toothpaste programmes as such intervention showed considerable effectiveness in reducing dental caries among six years old children, mainly in conflict zones [42, 43]. Unfortunately, dental caries and the promotion of healthy oral and eating behaviours are seldom in the WHO EMR, as this review clarified.

The limitations of the current review are that firstly, the evaluation of OHCS in the WHO-EMR has been conducted for a long time; secondly, the grey literature (respective countries' dental/medical councils, commission, societies, and Ministries information) has been searched, and unfortunately, information related to dental healthcare services are seldom available and highly ignored. Thirdly, no data about OHC responsiveness (person’s dignity, autonomy, confidentiality and/or satisfaction) were available in the included studies. However, Table 5 included the latest information reported by the WHO regarding oral and dental disease prevalence, challenges to dental care, and OHCS profile in the WHO-EMR.

## Conclusions

OHCS provision in the countries of the WHO-EMR region need better dental care prevention strategies. The high prevalence of dental diseases and resource deficiencies (OHCS expenditure is unknown) means MOHs struggle to meet populations’ dental care needs. Moreover, the current dependence of governmental OHCS primarily on basic dental treatment provisions and a scarcity of prevention programmes exacerbates the problem.

Therefore, further studies are needed to assess the OHCS performance, gaps, and effectiveness to predict the most effective and cost-effective OHCS practical preventive model. This model should tackle the seriously deteriorated oral health status in the region, to implement the most recent WHO recommendation and integrate OHCS as an essential part of Universal Health Coverage [44].

## Data Availability

All data generated or analysed during this study are included in this published article.

## Abbreviations

DAC: Development Assistance Committee
Dmft: Decay, missing and filled deciduous teeth
DMFT: Decayed, Missing and filled Permanent Teeth
IUHPE: International Union for Health Promotion and Education
LMICs: Low- and Middle-Income Countries
MOH: Ministry of Health
OHCS: Oral Health Care Services
UNHCR: United Nations Refugee Agency
UNICEF: United Nations Children's Fund
UNRWA: United Nations Relief and Works Agency for Palestine Refugees in the Near East
WHO: World Health Organization
WHO-EMR: WHO-Eastern Mediterranean Region
