# Correction: Provision of oral healthcare services in WHO-EMR countries: a scoping review

**DOI:** 10.1186/s12903-024-04702-y

**Published:** 2024-08-07

**Authors:** Lamis Abuhaloob, Celine Tabche, Federica Amati, Salman Rawaf

**Affiliations:** WHO Collaborating Centre for Public Health Education and Training, Primary Care & Public Health, School of Public Health, Faculty of Medicine, Imperial College London, The Reynolds Building, St. Dunstan’s Road, London, W6 8RP UK

**Correction to: BMC Oral Health (2024) 24:705**

10.1186/s12903-024-04446-9

In this article [1], the author of the article would like to remove the existing Acknowledgement text and replace with the below.
